# Retraction Note: High bioavailablilty iron maize (*Zea mays* L.) developed through molecular breeding provides more absorbable iron in vitro (*Caco-2* model) and in vivo (*Gallus gallus*)

**DOI:** 10.1186/s12937-015-0109-x

**Published:** 2015-12-29

**Authors:** Elad Tako, Owen A. Hoekenga, Leon V. Kochian, Raymond P. Glahn

**Affiliations:** USDA-ARS Robert W. Holley Center for Agriculture and Health, Cornell University, 538 Tower Road, Ithaca, NY 14853 USA

The authors are retracting this article [1] because the genetic makeup of the maize lines used in this study is not as shown in Fig. 1. It has been determined that the conclusion that the maize lines were near isogenic was incorrect; however, the rest of the data pertaining to the level of available iron in the various maize lines are correct. In view of this finding, the original claim that near isogenic maize lines have been created with reference to the QTL controlling the level of available iron in maize is no longer substantiated. This finding substantially alters the scientific value and impact of the article and warrants the article’s retraction in its entirety.

The authors wish to emphasize that the in vitro and in vivo methods and results presented in this article [1] were valid and legitimate. Clearly, these methods demonstrated that one line of maize was high in bioavailable Fe relative to the other. However, the unique aspect of this work was that the increased seed Fe bioavailability was associated with specific regions of the maize genome. This claim now has to be withdrawn due to the determination that the genotypes of the maize lines used in this study were not what was reported in Fig. 1.
